# The Usefulness of the Armchair Sign for the Diagnosis of Psychosomatic-Prone Myofascial Pain Syndrome in Patients with Incurable Cancer: A Secondary Analysis of a Prospective Multicenter Observational Clinical Study

**DOI:** 10.1089/pmr.2021.0033

**Published:** 2021-09-17

**Authors:** Hideaki Hasuo, Hiroto Ishiki, Yoshinobu Matsuda, Hiromichi Matsuoka, Shuji Hiramoto, Junya Kinkawa, Masanori Nojima

**Affiliations:** ^1^ Department of Psychosomatic Medicine, Kansai Medical University, Osaka, Japan.; ^2^ Department of Palliative Medicine, National Cancer Center Hospital, Tokyo, Japan.; ^3^ Department of Psychosomatic Medicine, National Hospital Organization Kinki-Chuo Chest Medical Center, Sakai, Japan.; ^4^ Department of Psycho-Oncology, National Cancer Center Hospital, Tokyo, Japan.; ^5^ Department of Clinical Oncology and Palliative Medicine, Mitsubishi Kyoto Hospital, Kyoto, Japan.; ^6^ Department of Rehabilitation, Medical Corporation Jinseikai, Chiba, Japan.; ^7^ Center for Translational Research, The Institute of Medical Science Hospital, The University of Tokyo, Tokyo, Japan.

**Keywords:** armchair sign, incurable cancer, myofascial pain syndrome, psychosomatic disease

## Abstract

***Background:*** Because psychosomatic diseases are pathological conditions, it is difficult to identify their degrees. The armchair sign is a test used to assess voluntary muscle relaxation.

***Objective:*** We aimed to evaluate the usefulness of the armchair sign for the diagnosis of psychosomatic-prone myofascial pain syndrome (MPS) in patients with incurable cancer.

***Design:*** This was a secondary analysis of a prospective multicenter observational clinical study.

***Setting/Patients:*** Patients with incurable cancer who were referred to palliative care services at five institutions in Japan between March 2018 and December 2018.

***Results:*** A total of 101 patients were enrolled, of whom 44 met MPS diagnostic criteria. Of these, 27 patients (61.3%) had psychosomatic-prone MPS. There was a significant association between the armchair sign and psychosomatic-prone MPS (*p* = 0.002). Sensitivity and specificity were 40.7% (95% confidence interval [CI]: 18.0–63.4) and 100.0%, respectively. The area under the curve score was 0.704 (95% CI: 0.553–0.855).

***Conclusions:*** The armchair sign may be useful as an ancillary test for the diagnosis of psychosomatic-prone MPS in patients with incurable cancer.

***Trial Registration:*** UMIN000031338. Registered February 16, 2018.

## Introduction

Psychosomatic disease is defined as any physical pathological condition with organic or functional damage that is affected by psychological factors during onset or development.^1^ Diagnosis of psychosomatic disease leads to psychological approaches in addition to physical approaches.^1^ Myofascial pain syndrome (MPS) is functional damage that presents with symptoms of muscle pain. MPS is found in 31%–45% of cancer patients who complain of pain.^2–4^ One of the reference criteria of the MPS diagnostic criteria is that pain worsens with stress.^5^ The relationship between low-back pain, such as myofascial pain, and psychological stress has been reported in a study that identified aspects of psychological stress-induced pain exacerbation.^6^ Furthermore, an observational study investigated psychosomatic-prone MPS in cancer patients, which showed that 57.1% of cancer patients with MPS experienced psychological stress.^4^ Psychosomatic-prone MPS in patients with incurable cancer is difficult to diagnose properly and may be diagnosed as psychogenic pain or cause opioid-induced delirium.^7^

Because psychosomatic diseases are pathological conditions, it is difficult to identify their degrees. Patients with advanced cancer indicated that the physical examination was a highly positive aspect of their care.^8^ These benefits are perceived as having both symbolic and pragmatic value.^8^ To be able to objectively evaluate degrees of psychosomatic disorders during physical examinations would be valuable. However, there were no reports that investigated such physical examinations. The armchair sign is a test to assess voluntary muscle relaxation, which, if positive, indicates insufficient relaxation. The armchair sign is rarely reported in patients with psychosomatic disorders such as chronic tension headache,^9^ whereas it is empirically used in psychosomatic medicine to assist in the diagnosis of psychosomatic diseases. The mechanism by which unconsciously sustained muscle tension under psychological stress leads to long-lasting pain and muscle tenderness has been demonstrated *in vivo*^10^; however, there have been no studies that have reported whether unconsciously sustained muscle tension under psychological stress is associated with voluntary muscle relaxation. We hypothesized that unconsciously sustained muscle tension under psychological stress, which is common in patients with incurable cancer, may make it difficult for muscles to relax. It would be valuable if the armchair sign has utility as an ancillary test for psychosomatic-prone MPS.

## Methods

### Objective

We aimed to evaluate the usefulness of the armchair sign for the diagnosis of psychosomatic-prone MPS in patients with incurable cancer.

### Study design

This study was a secondary analysis of a multicenter prospective observational study at five tertiary care centers in Japan between March 2018 and December 2018. This study received approval from the medical ethics committee of Kansai Medical University (reference number: 2017289).

### Study participants

Patients who met the following eligibility criteria were included in the study: (1) referred to a palliative care service, (2) informed of a malignancy diagnosis, (3) the malignant disease is incurable, (4) aged 20 years or older, and (5) average pain numerical rating scale over 24 hours before enrollment of 4 or higher. The exclusion criteria were any comorbidity relating to psychiatric diseases or conditions that made communication difficult (e.g., cognitive impairment or delirium). Informed consent was taken from all the patients.

### Data collection

Data on patient characteristics were recorded at enrollment. Data regarding MPS diagnosis of the painful sites on the posterior side of the body were collected when initiating palliative care. MPS was diagnosed according to the essential diagnostic criteria of Rivers et al.^5^ We determined the presence or absence of psychosomatic-prone MPS according to whether or not one of the reference diagnostic criteria of Rivers et al. (pain worsens with stress) was met.^5^ This was determined by palliative care physicians with >10 years of experience by asking patients, “Does your MPS pain worsen with stress?” Of the five tertiary care centers, two had oncology and three had psychosomatic medicine as subspecialties of the palliative care physicians.

**FIG. 1.**
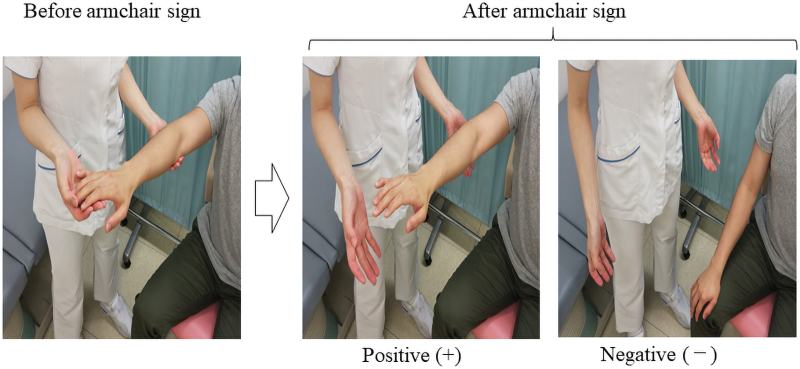
Evaluation of the armchair sign.

To perform the armchair sign, a physician asked the patient to raise one of the arms forward while supporting the arm with their hand and instructed the patient, “relax your arm and tell me when it is fully relaxed.” When the patient answered yes, the physician removed the supporting hand. If the arm of the patient was insufficiently relaxed, it did not fall completely. The test was considered positive (+) if the patient's arm was kept in the horizontal position, or negative (−) if the patient's arm fell completely (Fig. 1).

### Statistical analysis

Data are reported as means with standard deviations, or frequencies with 95% confidence intervals (CIs), as appropriate. Patients with incurable cancer were classified into two groups: psychosomatic-prone MPS and control (nonpsychosomatic-prone MPS) groups. We used unpaired *t*-tests and Fisher's exact tests to compare the dependent variables between the two groups.

Sensitivity, specificity, positive predictive value, and negative predictive value of the armchair sign with or without psychosomatic-prone MPS were calculated, on the basis of whether the armchair sign was negative (−). The receiver operating characteristic curve was calculated and the area under the curve (AUC) score was obtained.

## Results

A total of 541 patients were referred to palliative care services, and 101 patients met eligibility criteria. None of the patients met the exclusion criteria. Patient characteristics are given in Table 1.

**Table 1.** Demographic and Clinical Characteristics of Study Participants

Age (years), mean (SD)	60.8 (12.4)
Gender, *n* (%)	
Male	42 (41.6)
Female	59 (58.4)
Primary cancer site, *n* (%)	
Lung	31 (30.7)
Gastrointestinal	25 (24.7)
Liver, pancreas, biliary system	15 (14.8)
Gynecological	11 (10.9)
Head and neck	5 (5.0)
Others	14 (13.9)
ECOG PS, *n* (%)	
0–2	52 (51.5)
3–4	49 (48.5)
Anticancer treatment, *n* (%)	
No	51 (50.5)
Yes	50 (49.5)
Chemotherapy	45 (41.6)
Radiotherapy	15 (14.9)
Medical devices, *n* (%)	
No	65 (64.4)
Yes	36 (35.6)
Central venous catheter	2 (1.9)
Central venous port	15 (14.9)
Nasogastric tube	2 (1.9)
Nephrostomy catheter	2 (1.9)
Urethral catheter	5 (5.0)
Stoma	4 (4.0)
Others	6 (6.0)

ECOG PS, Eastern Cooperative Oncology Group performances status; SD, standard deviation.

Of the 101 enrolled patients, 44 met diagnostic criteria for MPS. On the basis of the criteria for diagnosis of MPS with and without psychological stress, study participants were classified into the psychosomatic-prone MPS (*n* = 27) or control group (*n* = 17). The proportion of psychosomatic-prone MPS patients among all MPS patients was 61.3% (95% CI: 43.8–78.8). There was no significant difference in proportion of patients between the two institutions with oncologists (58.8%, 95% CI: 34.7–82.9) and the three institutions with psychosomatic physicians (63.0%, 95% CI: 44.4–81.6; *p* = 0.515). Table 2 gives the demographic and clinical characteristics of both groups.

**Table 2.** Comparison Between Demographic Information, Clinical Characteristics, and Measures of the Psychosomatic-Prone Myofascial Trigger Syndrome and Control Groups

	Psychosomatic-prone MPS group (n = 27)	Nonpsychosomatic-prone MPS group (n = 17)	*p*
Age (years), mean (SD)	60.7 (12.6)	60.5 (12.6)	0.982
Gender (female), *n* (%)	14 (51.9)	10 (58.8)	0.405
ECOG PS, *n* (%)			
0–2	10 (37.0)	8 (47.1)	0.319
3–4	17 (63.0)	9 (52.9)	
Anticancer treatment (yes), *n* (%)	12 (44.4)	6 (35.3)	0.237
Medical devices (yes), *n* (%)	7 (25.9)	6 (35.3)	0.227
Site of MPS, *n* (%)			
Upper back	17 (63.0)	8 (47.1)	0.319
Lower back	10 (37.0)	9 (52.9)	
Pain NRS score of MPS, mean (SD)	60.7 (12.6)	60.9 (12.4)	0.188
Armchair sign, *n* (%)			
(+)	11 (40.7)	0 (0)	<0.001
(−)	16 (59.3)	17 (100.0)	

MPS, myofascial pain syndrome; NRS, numerical rating scale.

Of the 101 enrolled patients, 100 met the enforcement for armchair sign. The armchair sign was positive (+) in 25 patients and negative (−) in 75 patients. There was no significant association between armchair sign and all MPS (*p* = 0.594).

**FIG. 2.**
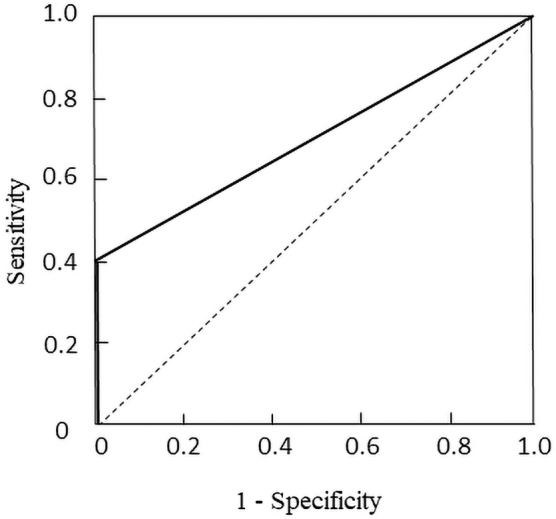
Receiver operating characteristic curve of the armchair sign with or without psychosomatic-prone myofascial pain syndrome.

In the 44 patients with MPS, the armchair sign was positive (+) in 11 patients and negative (−) in 33 patients. There was no significant difference in proportion of positive (+) between the two institutions with oncologists (23.5%, 95% CI: 2.7–44.3) and the three institutions with psychosomatic physicians (25.9%, 95% CI: 4.8–46.8; *p* = 0.862). There was a significant association between armchair sign and psychosomatic-prone MPS (*p* = 0.002). Sensitivity, specificity, positive predictive value, and negative predictive value were 40.7% (95% CI: 18.0–63.4), 100.0%, 100.0%, and 51.5% (95% CI: 34.7–68.3), respectively. AUC score was 0.704 (95% CI: 0.553–0.855; Fig. 2).

## Discussion

The important finding of this study was the possible association between armchair sign and psychosomatic-prone MPS in patients with incurable cancer, which demonstrated the potential clinical utility of the armchair sign for the diagnosis of psychosomatic-prone MPS. Based on the AUC score, the accuracy of the test was close to 1, which indicated that the test had high accuracy.^11^ In this case, specificity and positive predictive value were 100.0%, which suggested that the armchair sign of + was useful for diagnosing psychosomatic-prone MPS. The only clinical characteristic that showed a significant difference between the psychosomatic-prone MPS and control groups was the armchair sign, whereas the sensitivity and negative predictive value of the armchair sign were low, which suggested that the armchair sign of – was not reliable. Thus, our study suggests that the armchair sign may be useful as an ancillary test for the diagnosis of psychosomatic-prone MPS in patients with incurable cancer.

We did not find an association between armchair sign and all MPS in patients with incurable cancer. Specifically, there was no association between sustained muscle tension and difficulty in voluntary muscle relaxation. In addition to psychological stress, MPS is associated with physical stress, such as sustained muscle tension due to positional restriction or repetitive movements.^12,13^ Despite finding an association between sustained muscle tension and difficulty in voluntary muscle relaxation under psychological stress, we could not ascertain its mechanism. In patients with psychosomatic-prone functional somatic syndrome, a significant negative correlation has been shown between subjective physical tension under psychological stress and objective physiological indices.^14^ Constant feelings of high physical tension hinder the ability to feel sensations of relaxation.^14^ This unconscious sustained muscle tension under psychological stress may have resulted in insufficient voluntary muscle relaxation.

The study has several limitations. First, this study was a secondary analysis of a prospective observational study. Therefore, the sample size was not calculated specifically for the aims of this study and the sampling method was not justified. Second, because psychosomatic disorders are pathological conditions, it was difficult to identify its degree. Given that previous studies have not reported clear criteria (i.e., Rivers' criteria for MPS),^5^ we made a comprehensive judgment based on objective assessments of medical professionals and subjective assessments of patients. In this study, there was no significant difference in diagnoses between psychosomatic physicians who are skilled in diagnosing psychosomatic disorders and oncologists who are less skilled. Third, because there have only been a few studies conducted on the armchair sign, only limited comparisons can be made with other study findings, and discussions regarding the association between the armchair sign and psychosomatic-prone MPS are limited. Fourth, the criterion of age was 20 years or older, so it has not been generalized. This can be suspected in conclusion, as armchair sign may only be reliable or sensitive only to younger age groups. Finally, our study was a preliminary study. We will conduct studies in the future to assess whether armchair sign can be an ancillary test for the diagnosis of psychosomatic-prone patients and can correlate with psychological tests.

## Conclusions

The armchair sign may be useful as an ancillary test for the diagnosis of psychosomatic-prone MPS in patients with incurable cancer.

## Abbreviations Used

AUC: area under the curve
CI: confidence interval
ECOG PS: Eastern Cooperative Oncology Group performances status
MPS: myofascial pain syndrome
NRS: numerical rating scale
SD: standard deviation
